# Bis­(2-hy­droxy­eth­yl)ammonium 2-bromo­phenolate

**DOI:** 10.1107/S1600536812033752

**Published:** 2012-08-01

**Authors:** Kamentheren Padayachy, Manuel A. Fernandes, Helder M. Marques, Andreas Lemmerer, Alvaro S. de Sousa

**Affiliations:** a School of Chemistry, Molecular Sciences Institute, University of the Witwatersrand, Private Bag 3, Wits 2050, Johannesburg, South Africa

## Abstract

In the crystal structure of the 1:1 title salt, C_4_H_12_NO_2_^+^·C_6_H_4_BrO^−^, hydrogen-bonding inter­actions originate from the ammonium cation, which adopts a *syn* conformation. A *gauche* relationship between the C—O and C—N bonds of the 2-hy­droxy­ethyl fragments also facilitates O—H⋯O inter­actions of bis­(2-hy­droxy­eth­yl)ammonium cation chains to phenolate O atoms. The resulting double-ion chains along [100] are further linked by N—H⋯O inter­actions, forming chains parallel to [110].

## Related literature

For structures of related 2-haloethyl­ammonium salts and properties of these salts, see: Cody (1981 ▶); Cody & Strong (1980 ▶); Prout *et al.* (1988 ▶); Castellari & Ottani (1995 ▶); de Sousa *et al.* (2010*a* ▶,*b* ▶); Larsen *et al.* (2005 ▶); Mootz *et al.* (1989 ▶). For graph-set motifs, see: Bernstein *et al.* (1995 ▶).
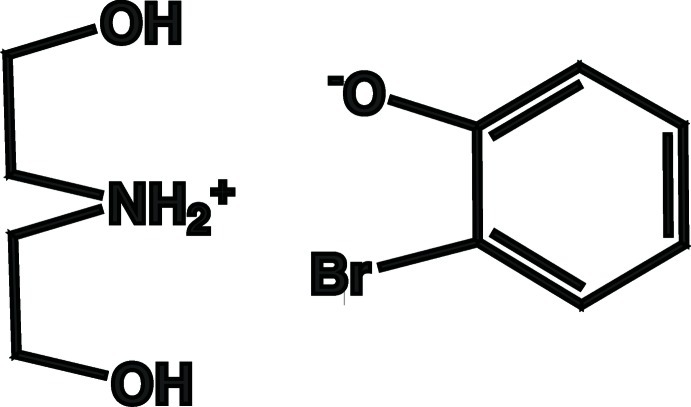


## Experimental

### 

#### Crystal data

C_4_H_12_NO_2_^+^·C_6_H_4_BrO^−^
*M* *_r_* = 278.15
Monoclinic, 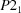

*a* = 8.0592 (1) Å
*b* = 7.6653 (1) Å
*c* = 9.7659 (2) Å
β = 107.250 (1)°
*V* = 576.16 (2) Å^3^
*Z* = 2
Mo *K*α radiation
μ = 3.56 mm^−1^
*T* = 173 K
0.48 × 0.21 × 0.20 mm

#### Data collection

Bruker APEXII CCD diffractometer
Absorption correction: gaussian (*XPREP*; Bruker, 2005 ▶) *T*_min_ = 0.280, *T*_max_ = 0.537
11689 measured reflections
2784 independent reflections
2638 reflections with *I* > 2σ(*I*)
*R*_int_ = 0.033

#### Refinement

*R*[*F* ^2^ > 2σ(*F* ^2^)] = 0.018
*wR*(*F* ^2^) = 0.042
*S* = 1.05
2784 reflections
149 parameters
1 restraint
H atoms treated by a mixture of independent and constrained refinement
Δρ_max_ = 0.17 e Å^−3^
Δρ_min_ = −0.28 e Å^−3^
Absolute structure: Flack (1983 ▶), 1290 Friedel pairs
Flack parameter: −0.012 (5)

### 

Data collection: *APEX2* (Bruker, 2005 ▶); cell refinement: *SAINT-NT* (Bruker, 2005 ▶); data reduction: *SAINT-NT*; program(s) used to solve structure: *SHELXS97* (Sheldrick, 2008 ▶); program(s) used to refine structure: *SHELXL97* (Sheldrick, 2008 ▶); molecular graphics: *PLATON* (Spek, 2009 ▶); software used to prepare material for publication: *WinGX* (Farrugia, 1999 ▶) and *PLATON*.

## Supplementary Material

Crystal structure: contains datablock(s) global, I. DOI: 10.1107/S1600536812033752/bh2446sup1.cif

Structure factors: contains datablock(s) I. DOI: 10.1107/S1600536812033752/bh2446Isup2.hkl

Supplementary material file. DOI: 10.1107/S1600536812033752/bh2446Isup3.cml

Additional supplementary materials:  crystallographic information; 3D view; checkCIF report

## Figures and Tables

**Table 1:** Hydrogen-bond geometry (Å, °)

*D*—H⋯*A*	*D*—H	H⋯*A*	*D*⋯*A*	*D*—H⋯*A*
O2—H2⋯O1	0.83 (2)	1.83 (2)	2.6393 (16)	165 (2)
O3—H3⋯O2^i^	0.84	1.87	2.7010 (18)	171
N1—H1*A*⋯O1^ii^	0.969 (18)	1.789 (19)	2.738 (2)	165.7 (16)
N1—H1*B*⋯O1	0.93 (2)	1.91 (2)	2.8259 (19)	169 (2)

Symmetry codes: (i) 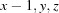; (ii) 
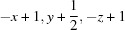
.

**Fig. 1.**
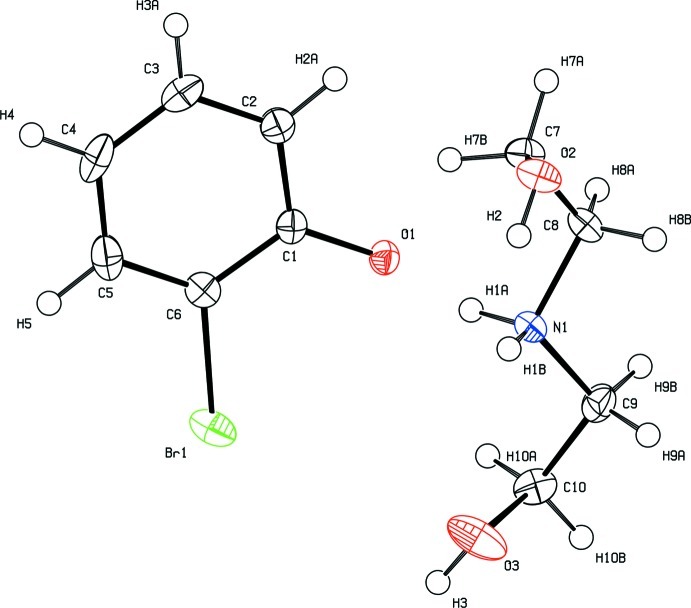
Molecular structure of the title compound, showing displacement ellipsoids at the 50% probability level.

**Fig. 2.**
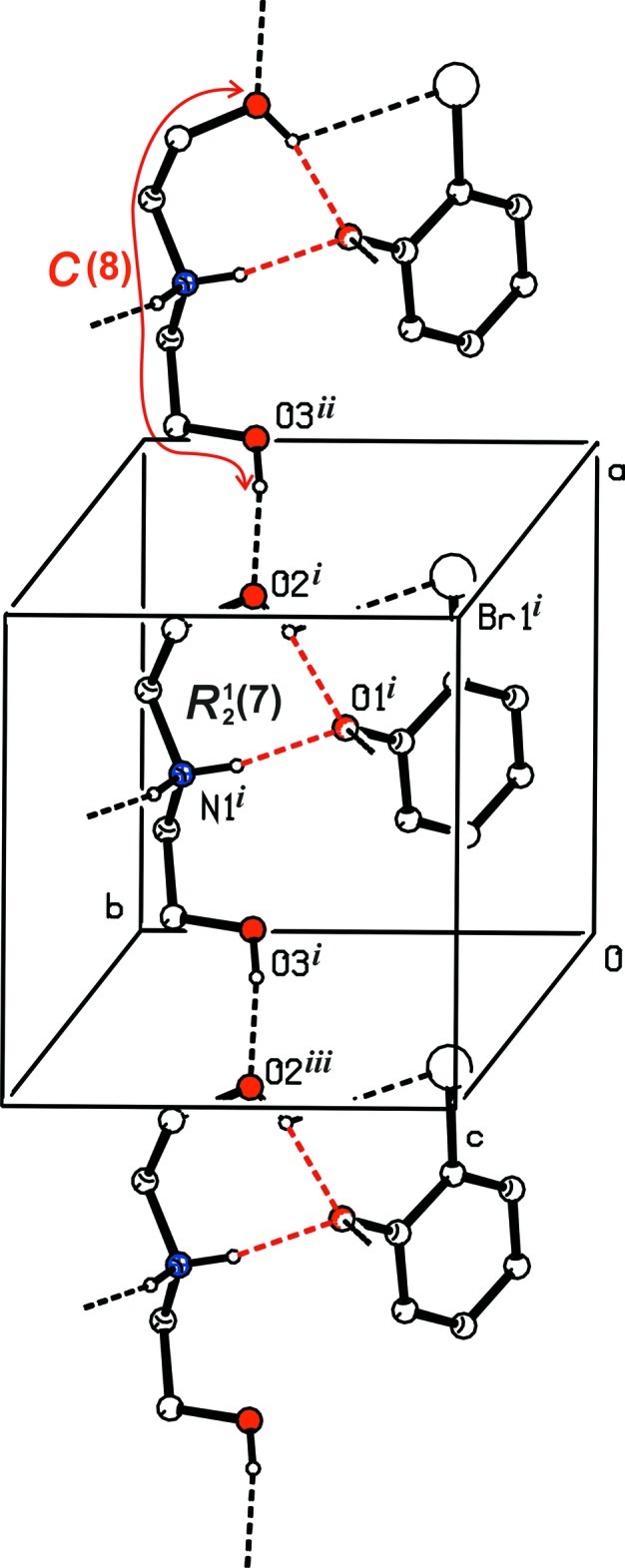
Chains along [100] defined by intermolecular O—H···O hydrogen bonds. Symmetry codes: (i) x, y, z; (ii) 1+x, y, z; (iii) -1+x, y, z.

**Fig. 3.**
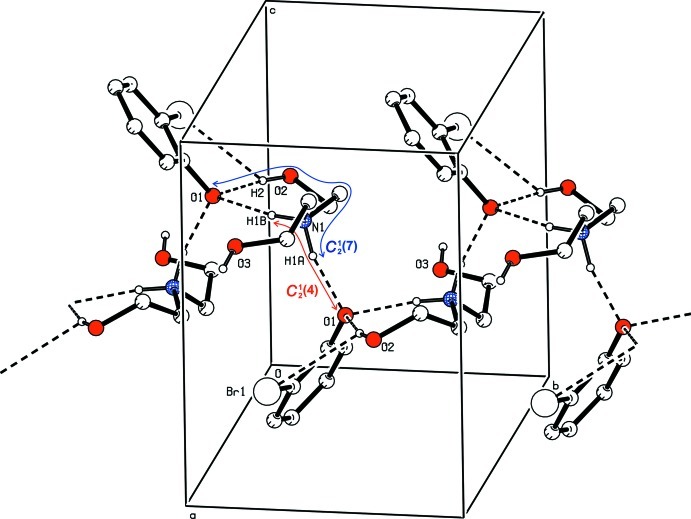
Layers parallel to (110) formed by hydrogen bonding double-ion chains along [100].

**Fig. 4.**
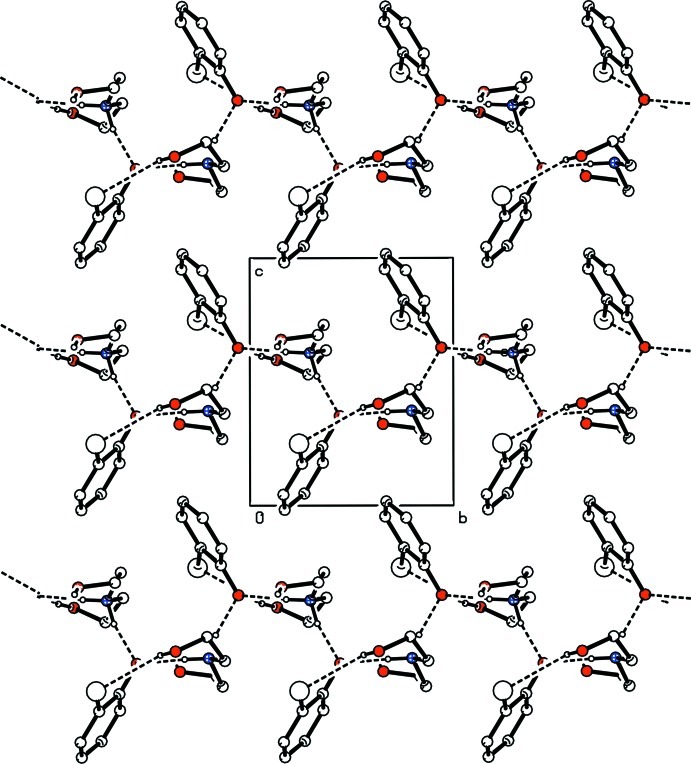
Binary C21(4) and C21(7) motifs along [010] described by N—H···O and O—H···O interactions originating at the bis(2-hydroxyethyl)ammonium cation.

## supplementary crystallographic information

### Comment

The molecular structure (Fig. 1) of the 1:1 salt of 2-bromophenol with diethanolamine (DEA) is reported. Our interest in studying DEA is aimed at developing amino alcohols as supramolecules in a crystal engineering strategy, for template self-assembly of these compounds in supramolecular structures. Directional hydrogen-bonding patterns associated with DEA have labeled this compound a potential supramolecule. It is known to aggregate into tubular columns (Mootz *et al.*, 1989); the behaviour is emulated by C— and N—alkylated derivatives (de Sousa *et al.*, 2010*a*,*b*). Specific O—H···O interactions of the 2-hydroxyalkyl groups contribute significantly towards tubular aggregation in alkylated derivatives of this compound. These hydrogen bonds also feature prominently in salts of DEA elucidating binding modes of thyroid hormones to transport proteins (Cody, 1981; Cody & Strong, 1980; Prout *et al.*, 1988); the template synthesis of heterometallic wheels (Larsen *et al.*, 2005); and in studies aimed at correlating structural and pharmacological properties of anti-inflammatory drugs (Castellari & Ottani, 1995).

O—H···O hydrogen bonds are highly influential in the molecular structure reported here for the 2-bromophenol (1:1) salt with DEA. The unitary level *C*(8) chain (Bernstein *et al.*, 1995), described by O3—H3···O2 hydrogen bonds along [100], defines the backbone of the crystal structure (Fig. 2 and Table 1). In these chains *syn* conformations of the bis(2-hydroxyethyl) ammonium cations enjoy *gauche* relationships between C—O and C—N bonds, enabling further O—H···O and N—H···O hydrogen bonds of this supramolecular synthon. Hydroxyethyl O atom O2 acts as a weakly bifurcated H-donor, *via* H2, to phenolate O1 and Br1 atoms (Table 1). The hydrogen bonding array of the double-ion pair is completed by the N1—H1B···O1 interaction involving the ammonium N1 atom acting as a hydrogen donor, *via* H1B, to the phenolate oxygen atom O1. The combined O2—H2···O1 and N1—H1B···O1 interactions describe a *R*_2_^1^(7) ring motif (Fig. 2) at the binary level (Bernstein *et al.*, 1995). Chains of double-ion pairs along [100] are linked by N1—H1A···O1 interactions (Table 1) to form layers parallel to the *ab* plane (Fig. 3). Within these layers N—H···O interactions define *C*_2_^1^(4) and *C*_2_^1^(7) motifs along [010] (Fig. 4) when combined with interactions N1—H1B···O1 and O2—H2···O1, respectively.

### Experimental

Diethanolamine (0.501 g, 4.8 mmol) was stirred in 20 ml of dimethylformamide. To this solution, sodium carbonate (0.504 g, 4.75 mmol) and 2-bromophenol (0.823 g, 4.76 mmol) was added with continuous stirring. The reaction mixture was allowed to stir for an additional 24 hours under ambient conditions. The mixture was filtered and the solvent removed under reduced pressure, to yield a clear viscous oil that crystallized upon standing.

### Refinement

Hydrogen atoms were visible in the difference maps, but those bonded to C atoms were positioned geometrically and refined as riding atoms, with C—H = 0.99 Å (CH_2_) or 0.95 Å (aromatic CH), as well as H3, bonded to O3, with O3—H3 bond length fixed to 0.84 Å. Isotropic displacement parameters for these H atoms were defined as *U_iso_*(H) = 1.2*U_eq_*(parent C atom) and *U_iso_*(H3) = 1.5*U_eq_*(O3). Other H atoms (H1A, H1B and H2), which are involved in hydrogen bonds, were refined freely.

### Crystal data

**Table:**

C_4_H_12_NO_2_^+^·C_6_H_4_BrO^−^	*F*(000) = 284
*M**_r_* = 278.15	*D*_x_ = 1.603 Mg m^−^^3^
Monoclinic, *P*2_1_	Mo *K*α radiation, λ = 0.71073 Å
Hall symbol: P 2yb	Cell parameters from 8333 reflections
*a* = 8.0592 (1) Å	θ = 2.2–28.2°
*b* = 7.6653 (1) Å	µ = 3.56 mm^−^^1^
*c* = 9.7659 (2) Å	*T* = 173 K
β = 107.250 (1)°	Needle, colourless
*V* = 576.16 (2) Å^3^	0.48 × 0.21 × 0.20 mm
*Z* = 2	

### Data collection

**Table:**

Bruker APEXII CCD diffractometer	2784 independent reflections
Radiation source: fine-focus sealed tube	2638 reflections with *I* > 2σ(*I*)
Graphite monochromator	*R*_int_ = 0.033
φ and ω scans	θ_max_ = 28.0°, θ_min_ = 2.2°
Absorption correction: gaussian (*XPREP*; Bruker, 2005)	*h* = −10→10
*T*_min_ = 0.280, *T*_max_ = 0.537	*k* = −10→10
11689 measured reflections	*l* = −12→12

### Refinement

**Table:**

Refinement on *F*^2^	Secondary atom site location: difference Fourier map
Least-squares matrix: full	Hydrogen site location: inferred from neighbouring sites
*R*[*F*^2^ > 2σ(*F*^2^)] = 0.018	H atoms treated by a mixture of independent and constrained refinement
*wR*(*F*^2^) = 0.042	*w* = 1/[σ^2^(*F*_o_^2^) + (0.025*P*)^2^] where *P* = (*F*_o_^2^ + 2*F*_c_^2^)/3
*S* = 1.05	(Δ/σ)_max_ = 0.001
2784 reflections	Δρ_max_ = 0.17 e Å^−^^3^
149 parameters	Δρ_min_ = −0.28 e Å^−^^3^
1 restraint	Absolute structure: Flack (1983), 1290 Friedel pairs
0 constraints	Flack parameter: −0.012 (5)
Primary atom site location: structure-invariant direct methods	

### Fractional atomic coordinates and isotropic or equivalent isotropic displacement parameters (Å^2^)

**Table:**

	*x*	*y*	*z*	*U*_iso_*/*U*_eq_	
C1	0.4737 (2)	0.35547 (19)	0.23985 (15)	0.0163 (3)	
C2	0.2924 (2)	0.3582 (2)	0.17355 (16)	0.0204 (3)	
H2A	0.2208	0.4246	0.2157	0.025*	
C3	0.2168 (2)	0.2669 (3)	0.04897 (18)	0.0278 (4)	
H3A	0.0942	0.2709	0.0076	0.033*	
C4	0.3155 (3)	0.1699 (2)	−0.01670 (18)	0.0299 (4)	
H4	0.2619	0.1080	−0.1028	0.036*	
C5	0.4937 (3)	0.1639 (2)	0.04447 (18)	0.0263 (4)	
H5	0.5637	0.0972	0.0009	0.032*	
C6	0.56985 (19)	0.2557 (3)	0.16984 (15)	0.0191 (3)	
O1	0.54554 (14)	0.44173 (13)	0.36173 (11)	0.0181 (2)	
Br1	0.815421 (18)	0.24105 (3)	0.250455 (16)	0.02891 (5)	
C7	0.7822 (2)	0.7869 (2)	0.46980 (18)	0.0222 (4)	
H7A	0.8807	0.8699	0.4908	0.027*	
H7B	0.7600	0.7583	0.5617	0.027*	
C8	0.6234 (2)	0.8745 (2)	0.37228 (18)	0.0210 (3)	
H8A	0.6257	0.9999	0.3971	0.025*	
H8B	0.6266	0.8654	0.2720	0.025*	
C9	0.3024 (2)	0.8634 (2)	0.26948 (17)	0.0235 (4)	
H9A	0.3174	0.9897	0.2557	0.028*	
H9B	0.2930	0.8035	0.1777	0.028*	
C10	0.1394 (3)	0.8345 (3)	0.3100 (2)	0.0235 (4)	
H10A	0.1458	0.8977	0.3998	0.028*	
H10B	0.0379	0.8786	0.2334	0.028*	
N1	0.4570 (2)	0.7953 (2)	0.38319 (17)	0.0168 (3)	
O2	0.83024 (16)	0.63195 (16)	0.41133 (14)	0.0241 (3)	
O3	0.1216 (2)	0.65362 (19)	0.3294 (2)	0.0379 (4)	
H3	0.0341	0.6357	0.3575	0.057*	
H1A	0.449 (2)	0.829 (2)	0.4768 (19)	0.017 (4)*	
H2	0.752 (3)	0.557 (3)	0.396 (2)	0.038 (6)*	
H1B	0.473 (3)	0.676 (3)	0.3791 (19)	0.012 (5)*	

### Atomic displacement parameters (Å^2^)

**Table:**

	*U*^11^	*U*^22^	*U*^33^	*U*^12^	*U*^13^	*U*^23^
C1	0.0227 (8)	0.0118 (7)	0.0152 (7)	−0.0009 (6)	0.0069 (6)	0.0028 (5)
C2	0.0243 (9)	0.0182 (7)	0.0196 (8)	0.0012 (6)	0.0077 (6)	0.0030 (6)
C3	0.0265 (8)	0.0303 (12)	0.0213 (7)	0.0000 (8)	−0.0008 (6)	0.0043 (7)
C4	0.0424 (11)	0.0284 (8)	0.0144 (8)	−0.0062 (8)	0.0016 (8)	−0.0026 (7)
C5	0.0398 (11)	0.0213 (8)	0.0217 (9)	0.0017 (7)	0.0150 (8)	−0.0010 (7)
C6	0.0211 (6)	0.0157 (8)	0.0219 (7)	−0.0016 (8)	0.0087 (5)	0.0026 (8)
O1	0.0192 (6)	0.0182 (5)	0.0170 (5)	−0.0016 (4)	0.0055 (4)	−0.0027 (4)
Br1	0.02163 (8)	0.02397 (8)	0.04356 (10)	0.00235 (9)	0.01340 (6)	−0.00094 (11)
C7	0.0176 (8)	0.0238 (10)	0.0252 (8)	−0.0027 (6)	0.0063 (6)	−0.0042 (6)
C8	0.0210 (8)	0.0180 (7)	0.0267 (8)	−0.0036 (6)	0.0111 (7)	0.0011 (6)
C9	0.0244 (9)	0.0243 (8)	0.0191 (8)	0.0031 (7)	0.0022 (7)	0.0042 (6)
C10	0.0195 (10)	0.0225 (9)	0.0273 (10)	0.0044 (7)	0.0050 (8)	−0.0048 (8)
N1	0.0162 (7)	0.0151 (6)	0.0201 (7)	−0.0009 (4)	0.0070 (5)	0.0006 (4)
O2	0.0187 (6)	0.0195 (6)	0.0356 (7)	−0.0023 (5)	0.0104 (5)	−0.0033 (5)
O3	0.0285 (9)	0.0231 (8)	0.0722 (12)	−0.0009 (6)	0.0306 (8)	−0.0068 (7)

### Geometric parameters (Å, º)

**Table:**

C1—O1	1.3344 (18)	C7—H7B	0.9900
C1—C6	1.403 (2)	C8—N1	1.505 (2)
C1—C2	1.411 (2)	C8—H8A	0.9900
C2—C3	1.379 (2)	C8—H8B	0.9900
C2—H2A	0.9500	C9—C10	1.496 (3)
C3—C4	1.378 (3)	C9—N1	1.496 (2)
C3—H3A	0.9500	C9—H9A	0.9900
C4—C5	1.383 (3)	C9—H9B	0.9900
C4—H4	0.9500	C10—O3	1.412 (2)
C5—C6	1.388 (2)	C10—H10A	0.9900
C5—H5	0.9500	C10—H10B	0.9900
C6—Br1	1.9041 (15)	N1—H1A	0.969 (18)
C7—O2	1.4204 (19)	N1—H1B	0.93 (2)
C7—C8	1.508 (2)	O2—H2	0.83 (2)
C7—H7A	0.9900	O3—H3	0.8400
			
O1—C1—C6	123.26 (14)	N1—C8—H8A	109.1
O1—C1—C2	121.22 (14)	C7—C8—H8A	109.1
C6—C1—C2	115.53 (14)	N1—C8—H8B	109.1
C3—C2—C1	121.58 (16)	C7—C8—H8B	109.1
C3—C2—H2A	119.2	H8A—C8—H8B	107.8
C1—C2—H2A	119.2	C10—C9—N1	110.82 (14)
C4—C3—C2	121.27 (17)	C10—C9—H9A	109.5
C4—C3—H3A	119.4	N1—C9—H9A	109.5
C2—C3—H3A	119.4	C10—C9—H9B	109.5
C3—C4—C5	119.06 (16)	N1—C9—H9B	109.5
C3—C4—H4	120.5	H9A—C9—H9B	108.1
C5—C4—H4	120.5	O3—C10—C9	108.25 (17)
C4—C5—C6	119.67 (16)	O3—C10—H10A	110.0
C4—C5—H5	120.2	C9—C10—H10A	110.0
C6—C5—H5	120.2	O3—C10—H10B	110.0
C5—C6—C1	122.89 (15)	C9—C10—H10B	110.0
C5—C6—Br1	117.92 (13)	H10A—C10—H10B	108.4
C1—C6—Br1	119.19 (12)	C9—N1—C8	111.74 (13)
O2—C7—C8	113.57 (13)	C9—N1—H1A	109.6 (10)
O2—C7—H7A	108.9	C8—N1—H1A	105.5 (10)
C8—C7—H7A	108.9	C9—N1—H1B	114.2 (12)
O2—C7—H7B	108.9	C8—N1—H1B	104.8 (14)
C8—C7—H7B	108.9	H1A—N1—H1B	110.6 (16)
H7A—C7—H7B	107.7	C7—O2—H2	111.9 (16)
N1—C8—C7	112.52 (13)	C10—O3—H3	109.5
			
O1—C1—C2—C3	−178.80 (15)	C2—C1—C6—C5	−0.6 (2)
C6—C1—C2—C3	0.6 (2)	O1—C1—C6—Br1	−0.1 (2)
C1—C2—C3—C4	−0.5 (3)	C2—C1—C6—Br1	−179.51 (11)
C2—C3—C4—C5	0.3 (3)	O2—C7—C8—N1	82.70 (18)
C3—C4—C5—C6	−0.3 (3)	N1—C9—C10—O3	−59.0 (2)
C4—C5—C6—C1	0.4 (3)	C10—C9—N1—C8	−160.34 (16)
C4—C5—C6—Br1	179.38 (13)	C7—C8—N1—C9	−170.68 (14)
O1—C1—C6—C5	178.81 (15)		

### Hydrogen-bond geometry (Å, º)

**Table:**

*D*—H···*A*	*D*—H	H···*A*	*D*···*A*	*D*—H···*A*
O2—H2···O1	0.83 (2)	1.83 (2)	2.6393 (16)	165 (2)
O3—H3···O2^i^	0.84	1.87	2.7010 (18)	171
N1—H1*A*···O1^ii^	0.969 (18)	1.789 (19)	2.738 (2)	165.7 (16)
N1—H1*B*···O1	0.93 (2)	1.91 (2)	2.8259 (19)	169 (2)
O2—H2···Br1	0.83 (2)	2.92 (2)	3.3690 (13)	115.6 (18)

Symmetry codes: (i) *x*−1, *y*, *z*; (ii) −*x*+1, *y*+1/2, −*z*+1.
